# MicroRNA Dysregulation in Epilepsy: From Pathogenetic Involvement to Diagnostic Biomarker and Therapeutic Agent Development

**DOI:** 10.3389/fnmol.2021.650372

**Published:** 2021-03-12

**Authors:** Jialu Wang, Jiuhan Zhao

**Affiliations:** Department of Neurology, First Affiliated Hospital of China Medical University, Shenyang, China

**Keywords:** microRNA, epilepsy, pathogenesis, biomarker, diagnosis, therapy

## Abstract

Epilepsy is the result of a group of transient abnormalities in brain function caused by an abnormal, highly synchronized discharge of brain neurons. MicroRNA (miRNA) is a class of endogenous non-coding single-stranded RNA molecules that participate in a series of important biological processes. Recent studies demonstrated that miRNAs are involved in a variety of central nervous system diseases, including epilepsy. Although the exact mechanism underlying the role of miRNAs in epilepsy pathogenesis is still unclear, these miRNAs may be involved in the inflammatory response in the nervous system, neuronal necrosis and apoptosis, dendritic growth, synaptic remodeling, glial cell proliferation, epileptic circuit formation, impairment of neurotransmitter and receptor function, and other processes. Here, we discuss miRNA metabolism and the roles of miRNA in epilepsy pathogenesis and evaluate miRNA as a potential new biomarker for the diagnosis of epilepsy, which enhances our understanding of disease processes.

## Introduction

Epilepsy is the result of a group of transient abnormalities in brain function caused by an abnormal, highly synchronous discharge of brain neurons. Its clinical manifestations mainly include recurrent convulsions and changes in consciousness, which seriously affect the work, daily activities, and physical and mental health of those afflicted with this disorder (Henshall, 2014). There are about 50,000,000 cases of epilepsy worldwide, with approximately 9,000,000 cases in China. Changes in multiple gene patterns in the brain result in the variations in cellular protein metabolism observed in the brain tissue of patients with epilepsy. The pathological mechanism of this disorder has not been clearly defined, and the processes related to neuronal apoptosis, glial regeneration, and the inflammatory response and the molecular mechanisms involved in the multiple links in the genetic information chain (e.g., gene translation, transcription, and post-transcription modification) require further investigation. Thus far, almost all transcriptional and post-transcriptional regulatory mechanisms have been shown to function abnormally during the onset of epilepsy (the process of a normal brain becoming epileptic), including classical transcription factors and epigenetic modifications (Becker et al., 2002; McClelland et al., 2011; Miller-Delaney et al., 2015; Brennan and Henshall, 2018).

MicroRNA (miRNA) is an endogenous non-coding gene that plays a role in post-transcriptional regulation and gene expression in advanced eukaryotes. Its main mode of action is to inhibit mRNA expression by identifying a complementary ribonucleotide sequence in the 3′-untranslated region (UTR) of the target messenger RNA (mRNA). Each miRNA may correspond to mRNAs encoded by hundreds of genes at the same time. Most miRNAs exhibit strictly regulated expression patterns, usually tissue-specific or even cell-specific, highlighting the importance of miRNAs in the time, space, and development stages of specific gene expression patterns. miRNAs can act as negative regulators of mRNAs that mediate gene expression (Uğurel et al., 2016). Therefore, the upregulation of miRNA may downregulate their target mRNA and the expression of the genetic information encoded by these mRNAs. The miRNAs of the nervous system form a complex gene regulatory network, which contains not only normal physiological regulatory information but also abundant neurobiological information related to neurological diseases (Christensen and Schratt, 2009).

In recent years, the emergence of gene chip technology has further explored the relationship between miRNA and epilepsy, as well as the treatment and prognosis of epilepsy (Henshall et al., 2016). Nervous system miRNAs are mainly involved in inflammatory responses, neuronal necrosis and apoptosis, dendritic growth, pathological circuit re-formation, glial cell proliferation, the formation of epileptic networks, impaired neurotransmitter release and receptor function. In short, a series of pathological changes in the nervous system eventually form a repeated excitatory cycle in the hippocampus, leading to the occurrence and development of epilepsy (Alsharafi et al., 2015; Pitkänen et al., 2016). In this review, the role of epilepsy-related pathological mechanisms and the regulatory involvement of miRNAs will be discussed to provide a new understanding of the early diagnosis and treatment of this disorder.

## miRNAS: Expression, Production and Mechanisms

MicroRNAs are small single stranded RNAs of about 22 nucleotides in length. miRNAs are widely distributed in animals, plants, fungi, and other multicellular eukaryotes. They are highly conserved in evolution and mainly located in non-coding regions of the genome. Although they do not encode proteins, miRNAs participate in important physiological and pathological processes. miRNAs can complement and pair with the 3′-UTR region of target gene mRNA, resulting in mRNA degradation or inhibition of translation. Thus, they can regulate up to 30% of protein expression after transcription (Bartel, 2004; He and Hannon, 2004; Bushati and Cohen, 2007).

Most miRNA promoters are recognized by RNA polymerase II (Pol II), and the initial miRNA transcription products must undergo splicing and polyadenylation (Winter et al., 2009). The initial transcription product, called primary miRNA (pri-miRNA), is about 1000 bp in size (Zeng et al., 2005). In the nucleus, the endonuclease RNase III-type protein Drosha cuts the double strand at the base of the pri-miRNA. The stem-loop intermediate with a phosphate group at the 5′-end of 60–100 bp and a dinucleotide overhang at the 3′-end is the precursor miRNA (pre-miRNA). Drosha is a non-specific RNase that cannot recognize pri-miRNA for specific cleavage and must form a complex with DGCR8 (Pasha) in animals. Specifically, the pri-RNA substrate is recognized by the double-stranded RNA binding site on DGCR8, and then Drosha cleaves the RNA 11 bp from the recognition point to generate the pre-miRNA (Chendrimada et al., 2005; Yeom et al., 2006). Next, Exportin-5, a transport protein on the nuclear membrane, binds to the pre-miRNA by recognizing the protruding dinucleotide structure at the 3′-end of the pre-miRNA, exporting it into the cytoplasm with the help of Ran-GTP. In the cytoplasm, the pre-miRNA is recognized by Dicer. The double strand of the spirochete is cut around the two helical corners from the stem-loop, resulting in a double-stranded RNA of 19–23 nucleotides that is similar in structure to small interfering RNA (siRNA). The mature miRNA comes from one arm of the pre-miRNA, and the other arm produces a fragment of the same length as the miRNA, namely miRNA^∗^. Finally, RNA helicase acts on the miRNA^∗^ duplex and undergoes a chain selection process. One strand of the miRNA^∗^ of the double RNA is degraded, and the other becomes the mature miRNA, which enters the RNA-induced silencing complex (RISC) (Chendrimada et al., 2007).

MicroRNAs are partially complementary to the 3′-UTR sequences of their target mRNAs. Target mRNA stability is not affected by the binding of miRNA; however, mRNA expression after translation initiation is inhibited. miRNAs can inhibit the extension or termination of translation or degrade newly synthesized peptide chains from ribosomes (Bazzini et al., 2012). Recent studies have shown that miRNAs are susceptible to various forms of RNA editing, including adenosine-to-inosine (A-to-I) RNA editing. The editing of miRNAs has a profound impact on the set of target genes that they can regulate. This type of modification can greatly expand the number of potential targets a single miRNA family can regulate and change our understanding of the role of miRNAs in homeostasis and disease environments (Zhang et al., 2006; Kawahara et al., 2007).

## The Role of miRNAs in Epilepsy Pathogenesis

**FIGURE 1:**
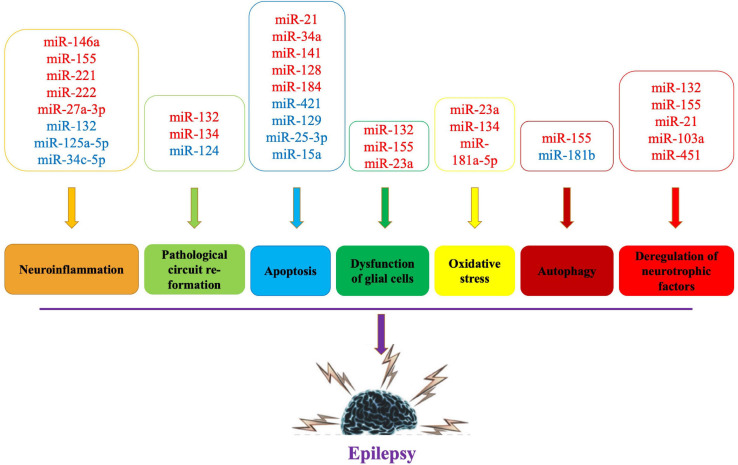
Pathogenic mechanisms and related miRNAs of epilepsy. Red words indicate the upregulated miRNAs and blue words indicate the downregulated miRNAs.

The exact etiology of epilepsy is still controversial. Its pathogenesis may be closely related to neuronal cell apoptosis, pathological circuit re-formation, glial fibroblast proliferation and inflammatory response (Chang and Lowenstein, 2003; Vezzani et al., 2011), and miRNAs may be involved in the occurrence and development of epilepsy by regulating these pathological processes (Cattani et al., 2016; Wang T. et al., 2017; Korotkov et al., 2020). The potential mechanisms of miRNA imbalance and the different roles of miRNAs in epilepsy pathogenesis are discussed below to highlight potential biomarkers and therapeutic developments (Figure 1).

### Neuroinflammation and miRNA

Cerebral tissue injury in patients with epilepsy can promote the release of inflammatory factors and induce inflammatory reactions. These inflammatory factors (e.g., interleukin-1 [IL-1], interferon-α [INF-α], and tumor necrosis factor-α [TNF-α]) can destroy the blood-brain barrier and aggravate damage to the nervous system and also excite neurons and promote repeated seizures (Vezzani et al., 2016, 2019; Rana and Musto, 2018).

In patients with drug-resistant epilepsy, miR-34c-5p was significantly downregulated, which might upregulate high mobility group protein (HMGB1) and IL-1β expression (Fu et al., 2020). Therefore, the authors of the study pointed out that decreased miR-34c-5p levels might exacerbate neuroinflammation in drug-resistant epilepsy and aggravate hippocampal neuron loss in epileptogenesis.

Toll-like receptor 4 (TLR4) is an important immune receptor involved in the development of epileptic inflammation by regulating the expression of nuclear factor-κB (NF-κB), tumor necrosis factor receptor-related factor 6, and IL-1 receptor-related kinase 1 (Kan et al., 2019). Elevated expression of IL-1, IL-6, and INF-α in epileptic foci demonstrates that inflammation is indeed closely related to the occurrence and development of epilepsy (Vezzani et al., 2008; Zhou et al., 2017). miR-146a regulates the expression of NF-κB, IL-1, and INF-α at the post-transcriptional level and affects the inflammatory reaction after an epileptic seizure. Increased miR-146a levels in the epileptic brain may alleviate inflammation, suggesting that miR-146a may be a target for disease treatment (Aronica et al., 2010; Tao et al., 2017; Zhang et al., 2018). In preclinical models of epilepsy, miR-146a also plays an important role in the TLR4 signaling pathway. After a seizure, TLR4 receptors are activated, and NF-κB enters the nucleus for activation. Activated NF-κB can upregulate the expression of miR-146a, IL-1, and INF-α. The increased miR-146a can suppress the activity of NF-κB, thereby reducing the production of IL-1 and INF-α and the inflammatory reaction caused by epilepsy (Boldin et al., 2011; Wang X. et al., 2018). In the refractory temporal lobe epilepsy (TLE) rat model, miR-146a increased the epilepsy susceptibility by reducing complement factor H. Thus, reducing the differential expression of miR-146a induced by epilepsy might reduce the occurrence of epilepsy (He et al., 2016). Enhanced miR-146a expression upregulated IL-1β in chronic TLE by downregulating complement factor H. Therefore, modulating the miR-146a-complement factor H-IL-1β loop circuit might be a novel therapeutic strategy for TLE (Li et al., 2018). miR-146a was up-regulated in the rat/mouse models. However, considering the differences in its downstream regulated target genes, miR-146a as a target for the treatment of epilepsy needs further study.

miR-155 is also associated with the regulation of inflammatory pathways in epilepsy. In children with chronic TLE, the expression levels of both miR-155 and TNF-α are increased. The increased TNF-α levels act as a feedback loop to regulate miR-155 expression. Thus, these two molecules interact to mediate the inflammatory process (Ashhab et al., 2013). Ashhab et al. (2013) confirmed that the expression levels of miR-155 and TNF-α were increased in children with chronic TLE, and miR-155 could increase TNF-α expression to enhance the inflammatory response.

The expression of miR-27a-3p is significantly increased in the hippocampus of epileptic rats (Lu et al., 2019). A miR-27a-3p inhibitor could effectively reduce IL-1β, IL-6, and TNF-α levels and neuronal apoptosis in the hippocampus of these rats (Lu et al., 2019). In contrast, expression of miR-125a-5p is downregulated in the hippocampus of pentylenetetrazol-induced epileptic rats (Liu Q. et al., 2019). Overexpression of miR-125a-5p attenuated epilepsy and decreased inflammatory factor levels in the hippocampus by suppressing calmodulin-dependent protein kinase IV (CAMK4), suggesting that miR-125a-5p might represent a novel treatment for epilepsy (Liu Q. et al., 2019). Lipopolysaccharide (LPS) treatment downregulated miR-132 levels *in vitro* (Ji et al., 2018). Overexpression of miR-132 reduced LPS-induced inflammatory injury, decreased the phosphorylated levels of kinases in the NF-κB and MEK/ERK pathways, and attenuated LPS-induced inflammatory cell injury by targeting tumor necrosis factor receptor-associated factor 6 (TRAF6) (Ji et al., 2018).

To conclude, dysregulated miRNA expression may be involved in epilepsy pathogenesis by regulating the expression of inflammatory factors (e.g., IL-1, INF-α, and TNF-α). Importantly, miR-146a may not only regulate inflammatory factors involved in the onset of epilepsy but may also be a biomarker for diagnosing epilepsy and an important therapeutic target.

### Apoptosis and miRNA

Recurrent epileptic seizures can cause neuronal apoptosis, and the decrease in cell number can reorganize the synapses between neurons and form abnormal synaptic loops that promote epilepsy recurrence (Henshall and Simon, 2005). Tivnan et al. (2011) were the first to demonstrate an association between miR-34 and apoptosis by reducing MAP3K9 mRNA and protein levels. After an epileptic seizure, the body upregulates the expression of pro-apoptotic miRNA and downregulates the expression of anti-apoptotic miRNA to increase cell apoptosis, which affects the occurrence and development of epilepsy (Hu et al., 2012).

miR-21 can inhibit cell apoptosis (Wang K. et al., 2017). miR-21 expression in the hippocampus is increased several hours after a seizure, which might reduce the inhibitory effect on the 3′ UTR region of the neurotrophin-3 and promote cell apoptosis (Peng et al., 2013). Risbud et al. (2011) demonstrated that the hippocampal miR-21 levels increased significantly from 2 days to 3 weeks in epileptic rats. They also found that the active protein of caspase-3 related to apoptosis signal transmission and the number of apoptotic cells also increased. Therefore, the mechanism underlying the role of miR-21 in epilepsy may be by activating pro-apoptotic genes to promote neuronal apoptosis.

miR-34a is an evolutionarily conserved pro-apoptotic miRNA that can be upregulated by activated p53. Hu et al. (2012) reported that miR-34a expression levels are increased in post-status epileptic rats. They showed that miR-34a was upregulated during seizure-induced neuronal death or apoptosis, and targeting miR-34a was neuroprotective and abrogated the increase in activated caspase-3 protein. miR-141 expression is also upregulated in patients with epilepsy. *In vitro*, miR-141 overexpression induces nerve cell apoptosis, suppresses proliferation, induces caspase-3/9, Bax and p53 expression, and reduces silent information regulator 1 protein expression (Liu D. et al., 2019). miR-128 expression is increased in rats with lithium chloride-induced epilepsy. Chen et al. (2019) demonstrated that miR-128 overexpression promoted nerve cell apoptosis, increased p53, Bax, and cytochrome c protein expression, and enhanced caspase-3/9 activity. Therefore, anti-miR-128 may be neuroprotective against epilepsy through the SIRT1/p53/caspase signaling pathway.

In addition to the role of pro-apoptotic miRNAs in epilepsy, some anti-apoptotic miRNAs are also differentially expressed and involved in the pathological mechanism of epilepsy. miR-184 is an apoptosis regulator that is upregulated in the CA3 subfield of the mouse hippocampus (McKiernan et al., 2012). McKiernan et al. (2012) showed that miR-184 had anti-apoptotic effects, which might be achieved by regulating the Numblike gene. miR-421 is downregulated in hippocampal neurons of epileptic mice. MYD88 is a target of miR-421. This miRNA could inhibit apoptosis and autophagy in hippocampal neurons in epileptic mice by downregulating the TLR/MYD88 pathway (Wen et al., 2018).

miR-129 expression is decreased in hippocampal neurons in epileptic rats (Wu et al., 2018). Wu et al. (2018) suggested that c-Fos was a potential target gene for miR-129, which could inhibit proliferation and apoptosis of hippocampal neurons in rats by repressing c-Fos expression through inhibiting the MAPK signaling pathway.

miR-25-3p is associated with oxidative stress and apoptosis. Li et al. (2020) found that miR-25-3p was downregulated concomitant with upregulated OXSR1 expression in the hippocampus of KA-treated rats. miR-15a expression is also downregulated in TLE tissues. Upregulated miR-15a significantly suppresses the apoptosis rate in epileptic cells. In addition, Fan et al. (2020) demonstrated that miR-15a directly targets GFAP. Thus, miR-15a upregulation might inhibit cell apoptosis and the inflammation in TLE by targeting GFAP, providing a potential therapeutic target for the treatment of TLE.

In general, the available data suggest that different miRNAs participate in epilepsy pathogenesis or protect neurons by regulating the level of apoptosis. Modulation of these differentially expressed miRNAs may provide new strategies for the treatment of epilepsy.

### Glial Cell Dysfunction and miRNA

Glial cells have supportive and protective effects on neurons, and they are mainly involved in the material metabolism of neurons. Their dysfunction may be an important cause of epilepsy. For example, dysfunction of glial cells may interfere with glutamate homeostasis and uptake, resulting in overexcitation of neurons and, eventually, epilepsy (Jimenez-Mateos et al., 2015). Reactive glial cells are also believed to contribute to the development of epilepsy by regulating brain inflammation and extracellular matrix (ECM) remodeling (Korotkov et al., 2020). miR-132 is one of the most commonly upregulated miRNAs in animal TLE models. Korotkov et al. (2020) demonstrated that the miR-132 expression is increased in the human epileptogenic hippocampus, particularly in glial cells. By transfecting miR-132 into human primary astrocytes, the expression of pro-epileptogenic COX-2, IL-1β, TGF-β2, CCL2, and MMP3 were decreased, suggesting that modulating miR-132 expression in astrocytes might be a potential therapeutic target warranting further investigation (Korotkov et al., 2020).

miR-155 is abundantly expressed in glial cells, and its expression is significantly increased in the brain tissue of epilepsy patients and kainate (KA)-induced epileptic mice. Fu et al. (2019) noted that abnormal proinflammatory cytokine expression and microglia morphology could be changed by silencing miR-155. In addition, a miR-155 antagomir may reduce microglia-impaired neuron excitability and attenuate KA-induced epilepsy by inhibiting microglia activation (Fu et al., 2019). Moreover, inhibition of miR-155 could also attenuate MMP3 overexpression after IL-1β stimulation in astrocytes, suggesting a possible strategy to prevent epilepsy via the modulation of glial cells and reduction of inflammation (Korotkov et al., 2018).

miR-23a is another miRNA for which increased expression is observed in the hippocampus of epileptic rats. Song et al. (2011) demonstrated that miR-23a had an effect on glial cell apoptosis. When miR-23a was inhibited, the expression of its target genes (STK4 and caspases) was increased, leading to increased glial cell apoptosis. Therefore, miR-23a might participate in the development of epilepsy by promoting glial cell proliferation and blocking glial cell apoptosis (Song et al., 2011).

### Pathological Circuit Re-formation and miRNA

The synapse is a special structure composed of the neuronal cell body, axon, and dendrite. An external signal stimulates the growth of dendritic spines and synapse formation. Pathological circuit re-formation refers to abnormal synaptic connections caused by brain tissue damage. The pathological neural circuit formed by this remodeling may lead to recurrent epilepsy (Janz et al., 2017). Previous studies have suggested that some miRNAs affect neuron development and pathological circuit re-formation by regulating protein synthesis, covalently modifying existing proteins, and reusing membrane receptors after epileptic seizures (Cattani et al., 2016).

miR-132 is involved in pathological circuit re-formation and the regulation of dendritic spines. Studies have also found that miR-132 expression is increased in children with TLE, speculating that increased miR-132 levels might affect pathological circuit re-formation and neuronal apoptosis through the regulation of P250GAP and promote the occurrence of epilepsy (Jimenez-Mateos et al., 2011; Scott et al., 2012). This miRNA was first discovered by Nudelman et al. (2010), who observed that the upregulation of miR-132 expression could increase neuronal activity in a rat model of pilocarpine-induced epilepsy. Through a series of complex signal transduction, miR-132 could inhibit the production of GTPase activating protein (P250GAP), thereby affecting the pathological circuit re-formation of hippocampal neurons (Nudelman et al., 2010). Moreover, increasing miR-132 expression in hippocampal neurons could induce pathological circuit re-formation, while inhibiting its expression would have the opposite effect (Wayman et al., 2008).

miR-124 is a brain-specific miRNA that was originally believed to be a key regulator of neuronal differentiation and nervous system development. It is extremely abundant in the brain and can regulate the growth of neuronal synapses. miR-124 also plays an important role in epilepsy. Wang W. et al. (2016) found that miR-124 expression was decreased in patients with epilepsy and rats after drug induced-seizures. CAMP response element-binding protein1 (CREB1) is a key regulator in epileptogenesis (Wang W. et al., 2016). miR-124 directly targets the 3′ UTR of the CREB1 gene to repress CREB1 expression and abrogate the-epileptic effect (Wang W. et al., 2016). The EPAC protein is a guanine nucleotide exchange factor that acts as an intracellular receptor for cyclic adenylate. Yang et al. (2012) found that a mutation in the mouse EPAC gene caused abnormal synaptic transmission, which reduced the spatial and social learning ability of mice. However, the silencing of miR-124 restored the cognitive function of the mice, suggesting that miR-124 was closely associated with pathological circuit re-formation and could further affect the cognitive function of epileptic mice (Yang et al., 2012).

miR-134 is a brain-specific, activity-regulated miRNA that has been implicated in the control of dendritic spine morphology (Jimenez-Mateos et al., 2012). miR-134 expression is upregulated in experimental epilepsy models and the human disease. Inhibition of miR-134 expression could reduce the number of neuronal spines and degree of pathological damage to brain tissue after an epileptic seizure (Jimenez-Mateos et al., 2012).

### Autophagy and miRNA

The relationship between autophagy and epilepsy has not been fully clarified. Some studies suggest that autophagy may induce epilepsy through the mammalian target of rapamycin (mTOR) pathway or abnormal glycogen accumulation. Some miRNAs may affect the onset of epilepsy by regulating the process of autophagy. For example, miR-34a negatively regulates autophagy and is up-regulated after epileptic status. However, autophagy is activated after recurrent neonatal convulsions, suggesting that miR-34a may play an important role in the excitatory toxicity induced by neonatal convulsions (Gan et al., 2015).

miR-155 induces autophagy through mTOR, and this effect is more obvious in the young mouse status epilepticus model, which strongly suggests the existence of this hypothetical pathway in epilepsy (Wan et al., 2014).

miR-181b expression is decreased in juvenile KA-induced epileptic rats (Wang et al., 2019). Wang et al. demonstrated that TLR4 is a direct target of miR-181b. This miRNA can inhibit the P38/JNK signaling pathway by targeting TLR4, thereby attenuating autophagy of KA-induced epileptic juvenile rats (Wang et al., 2019).

Furthermore, as mentioned above, miR-421 could inhibit the apoptosis and autophagy of hippocampal neurons in epilepsy mice by down−regulating the TLR/MyD88 pathway (Wen et al., 2018).

### Oxidative Stress and miRNA

Oxidative stress caused by excessive free radical release is involved in the pathological processes of many neurodegenerative diseases. However, the relationship between oxidative stress and epilepsy has only recently been recognized. Accumulating evidence demonstrates that oxidative stress is a key factor in not only the consequences of epilepsy but may also be involved in the disease pathogenesis. An impaired antioxidant system, mitochondrial dysfunction, and activation of the arachidonic acid pathway may be the main underlying causes of epilepsy pathogenesis. Oxidative stress affects the expression levels of multiple miRNAs, and, conversely, miRNAs could regulate many genes involved in the oxidative stress response. Both oxidative stress and miRNA regulatory networks influence processes of neurological diseases, including epilepsy (Konovalova et al., 2019).

miR-23a is one of the most common miRNAs involved in hippocampal neuronal injuries and spatial memory impairment in an experimental model of TLE. Zhu et al. (2019) found that miR-23a was upregulated in the hippocampus after status epilepticus (SE) in KA-induced TLE mice. In addition, this change in miR-23a expression was accompanied by hippocampal oxidative damage. Furthermore, hippocampal oxidative stress and neuronal injuries could be significantly improved by inhibiting miR-23a expression with miR-23a antagomirs. Thus, targeting miR-23a in the epileptic brain might provide a novel strategy for protecting against hippocampal neuronal injuries in TLE patients (Zhu et al., 2019).

Previous studies revealed the neuroprotective effect of miR-134 antagomirs, which could reduce ischemic injury and cause prolonged seizure suppression (Huang et al., 2014). It was reported that miR-134 levels were significantly upregulated in rat brain after KA-induced SE (Gao et al., 2019). A miR-134 antagonist could suppress lesion-induced endoplasmic reticulum stress and apoptosis-related CHOP expression. Gao et al. (2019) suggested silencing of miR-134 could modulate the epileptic phenotype by upregulating CREB, which might be a promising intervention for the treatment of epilepsy.

The miR-181a-5p expression levels are increased in a lithium-pilocarpine model of epilepticus in immature rats. Inhibition of miR-181a-5p might protect the hippocampus against the damage from an epileptic seizure through various mechanisms, including oxidative stress. Moreover, inhibition of miR-181a-5p could exert a seizure-suppressing effect via SIRT1 upregulation, suggesting a potential role for the miR-181a-5p/SIRT1 pathway in the development of temporal lobe epilepsy (Kong et al., 2020).

### Deregulation of Neurotrophic Factors and miRNA

Brain-derived neurotrophic factor (BDNF) and its receptor tropomyosin-related kinase B (TrkB) are involved in the pathophysiology observed with epilepsy. In recent years, the miRNAs that may be involved in BDNF-mediated epilepsy have received increasing attention. miR-155 expression levels were higher in epilepsy patients compared to the normal controls. Moreover, Duan et al. (2018) also demonstrated that miR-155 contributes to the occurrence of epilepsy through the PI3K/Akt/mTOR signaling pathway. Xiang et al. (2015) found a dramatic upregulation of miR-132 and BDNF mRNA expression in the hippocampal neuronal culture model of SE. In addition, their results suggested that miR-132 promotes epileptogenesis by regulating BDNF/TrkB signaling. In contrast, neurotrophin-3 mRNA levels decrease in the hippocampus following SE, concurrent with an increase in miR-21. Thus, the miR-21 levels in cultured hippocampal neurons are inversely correlated with neurotrophin-3 mRNA levels, and miR-21 is a candidate for regulating neurotrophin-3 signaling in the hippocampus following status epilepticus (Risbud et al., 2011).

miR-103a expression is increased in an epileptic rat model induced by lithium chloride-pilocarpine treatment. miR-103a inhibitors induced BDNF expression, increased the number of surviving neurons, and decreased the number of apoptotic neurons (Zheng et al., 2019). miR-451 is also upregulated in KA-induced epilepsy models, and miR-451 knockout improved the pathological changes in the hippocampus. In addition, miR-451 knockout might inhibit the apoptosis of hippocampal neurons. Glial cell line-derived neurotrophic factor (GDNF) is a target gene of miR-451. GDNF overexpression reversed the effect of miR-451 on KA-induced brain injury and neuronal apoptosis (Weng et al., 2020).

## miRNAs as Biomarkers of Epilepsy

For most epilepsy patients, clinicians can give a timely and correct diagnosis through patient history and clinical manifestations. Effective biomarkers can help to make the correct diagnosis and epilepsy classification and provide an opportunity to develop targeted therapy for epilepsy. Genetic biomarkers, such as the gamma-aminobutyric acid (GABA) receptor gene, 5-hydroxy tryptamine (5-HT) receptor gene, sodium channel voltage-gated type I-alpha (SCN1A) gene, aquaporin-4 (AQP4), and inwardly rectifying potassium channel (Kir4.1) gene, and inflammatory biomarkers (e.g., IL-2, IL-6, and TNF-α) may offer help in diagnosing epilepsy (Symonds et al., 2017). However, the application of these biomarkers is limited as some results are inconsistent and lack diagnostic specificity.

MicroRNA can affect the synthesis and molecular structure of a variety of proteins, and changes in miRNA expression levels and activity may affect cellular functions (Krol et al., 2010). Indeed, miRNA expression via oligonucleotides can easily lead to widespread gene expression changes (Bajan and Hutvagner, 2020). These properties make miRNAs useful epilepsy biomarkers and potential new therapeutic targets (Supplementary Table 1). Avansini et al. (2017) performed high-throughput sequencing analysis on plasma miRNA from 14 mesial TLE (MTLE) and 13 focal cortical dysplasia (FCD) samples along with 16 normal controls. They found that miR-134 was significantly downregulated in the plasma of MTLE patients, suggesting that decreased hsa-miR-134 expression could be a potential non-invasive biomarker to support the diagnosis of patients with MTLE. Other potential circulating biomarkers are miR-145, miR-181c, miR-199a, and miR-1183, which were overexpressed in the blood of patients with MTLE with hippocampal sclerosis (MTLE-HS) (Antônio et al., 2019). Serum miR-328-3p is also an important peripheral biomarker for the diagnosis of MTLE-HS with high area under the curve (AUC) values when comparing controls to Engel I (90.3%). For predicting the surgical prognosis of MTLE-HS patients, miR-654-3p had statistical power as a peripheral biomarker (AUC = 73.6%) to differentiate Engel I from Engel III-IV patients (Ioriatti et al., 2020). Wang et al. (2015b) used Illumina HiSeq2000 sequencing to screen for differentially expressed miRNAs in the serum of 30 epilepsy patients and 30 healthy controls. They found that miR-106b-5p had the highest diagnostic value for epilepsy, with a sensitivity of 80.3% and a specificity of 81.2%, suggesting that miR-106b-5p could be used as a non-invasive diagnostic biomarker for epilepsy (Wang et al., 2015b). An et al. (2016) recruited 90 epilepsy patients (57 cases of generalized seizures, 33 cases of focal seizures) and 90 healthy controls for their study that used a PCR method to detect the expression levels of four epilepsy-related miRNAs (miR-106b, miR-146a, miR-194-5p, and miR-301a) in serum. Compared to the control group, serum miR-106b, miR-146a, and miR-301a were significantly upregulated in the epilepsy group, while miR-194-5p was significantly downregulated. In addition, serum miR-106b and miR-146a expression levels were positively correlated with the severity of epilepsy. The combined detection of these two miRNAs in serum had better sensitivity and specificity for the prediction of epilepsy (An et al., 2016).

miR-129-2-3p is upregulated in the temporal cortex and plasma of patients with refractory TLE (Sun Y. et al., 2016). With increasing epilepsy frequency, miR-129-2-3p expression levels are also upregulated, and the prognosis of patients with epilepsy is also poor. Therefore, plasma miR-129-2-3p may be used as a potential non-invasive biomarker for early detection and clinical prognosis evaluation for refractory TLE (Sun Y. et al., 2016). In contrast, miR-145-5p expression levels in plasma are significantly downregulated in patients with refractory TLE. This decreased expression is positively correlated with the age of onset and frequency of epilepsy (Shen et al., 2019). Sun J. et al. (2016) found that the expression levels of miR-30a, miR-378, miR-106b, and miR-15a in the serum of patients with epilepsy were upregulated compared to the levels observed during the inter-seizure period. Among these miRNAs, miR-30a was positively correlated with seizure frequency but had no significant correlation with sex, age, and medical history (Sun J. et al., 2016). miR-4521 is upregulated in the brain tissue and serum of refractory epilepsy patients. Serum miR-4521 levels may represent a potential diagnostic biomarker for FCD with refractory epilepsy (Wang X. et al., 2016). Another study with FCD patients found that the expression of miR-323a-5p was significantly elevated in the cortex and plasma of FCD patients with refractory epilepsy, suggesting that abnormal miR-323a-5p expression could be used to monitor treatment responses in patients with FCD (Che et al., 2017). Serum of miR-146a and miR-155 levels are also significantly upregulated in genetic generalized epilepsy patients. Martins-Ferreira et al. (2020) suggested that the combined serum levels of miR-132, miR-146a, and miR-155 could discriminate between genetic generalized epilepsy patients and controls with high specificity and sensitivity.

Some circulating miRNAs have been associated with drug-resistant epilepsy. Wang et al. (2015a) used Illumina HiSeq2000 sequencing technology to analyze the differential expression of serum miRNAs in 30 drug-resistant epilepsy patients and 30 drug-sensitive epilepsy patients. miR-301a-3p is the most valuable biomarker for the identification of drug-resistant epilepsy to date. Multiple regression analysis showed that downregulated miR-301a-3p expression represents a potential biomarker for the diagnosis of drug-resistant epilepsy, with a sensitivity of 81.5% and specificity of 81.2% (Wang et al., 2015a). Leontariti et al. (2020) demonstrated that miR-134 and miR-146a serum levels were elevated in patients with drug-resistant epilepsy. These levels represented a significantly higher risk of developing drug-resistant epilepsy.

So far, new biomarkers for the diagnosis of epilepsy are still being evaluated. The expression changes of various miRNAs identified by expression profiling of circulating miRNA have been confirmed in epilepsy patients (Antônio et al., 2019; Brennan et al., 2020). There is evidence that epilepsy is associated with the expression changes observed in the circulating miRNAs. The inclusion of more cases and consistent studies of circulating miRNA detection techniques could enhance the potential of using miRNAs as biomarkers for epilepsy.

## miRNA-Based Therapeutic Approaches for Epilepsy

With the continuous deepening of the research on the mechanism of miRNAs involved in epilepsy pathogenesis, the idea of miRNA-targeted intervention to prevent or delay the occurrence of epilepsy is valuable. Because a single miRNA can simultaneously regulate multiple pathways, targeting a single miRNA may affect many cell processes and, thus, be an effective intervention strategy following epileptogenic injury. Many preclinical studies have demonstrated the function of miRNAs and their potential to treat acute or chronic epilepsy. The path of clinical transformation has begun. So far, miRNA-based therapies have been well-tolerated and have yielded therapeutic effects in preclinical studies (Supplementary Table 2).

Vagus nerve stimulation (VNS) has proven to be a safe and effective treatment for refractory epilepsy. This procedure could activate neuronal and astrocyte a7nAchR and inhibit the apoptotic and oxidant stress responses. Jiang et al. (2015) suggested that miR-210 plays an important role in the antioxidant stress and anti-apoptosis responses induced by VNS, indicating that the miR-210 is a potential mediator of VNS-induced neuroprotection against I/R injury. miR-137 is an extremely rich miRNA in the central nervous system and is believed to be closely associated with synaptic plasticity. In the pilocarpin-induced epileptic mouse model, miR-137 overexpression induced by intrahippocampal injection of a specific Agomir prolonged the latency period of spontaneously recurring seizures and reduced the severity of epilepsy (Wang W. et al., 2018). miR-135a silencing in an experimental temporal lobe epilepsy model reduced seizure activity at the spontaneous recurrent seizure stage by regulating Mef2 proteins, which are key regulators of excitatory synapse density (Vangoor et al., 2019). By Nissl staining, miR-134 silencing significantly reduced the loss of CA3 pyramidal neurons and abnormal mossy fiber germination. In addition, EEG and behavioral analysis showed that miR-134 antagonists had a palliative effect on experimental epileptic seizures. These results suggested that silencing miR-134 regulated epileptic phenotypes by upregulating its target gene CREB (Gao et al., 2019).

Silencing miR-132 inhibited the aberrant formation of dendritic spines and chronic spontaneous seizures in a lithium-pilocarpine-induced epileptic mouse model (Yuan et al., 2016). Experiments with cultured epileptic neurons suggesting that miR-132 silencing exerted a neuroprotective effect through the miR-132/p250GAP/Cdc42 pathway (Yuan et al., 2016). miR-204 directly targets and downregulates TrkB protein in various diseases (Xiang et al., 2016). Xiang et al. (2016) suggested that miR-204 overexpression caused anti-epileptogenic effects by regulating TrkB and its downstream ERK1/2-CREB signaling pathway. Moreover, Zheng et al. (2016) demonstrated that miR-219 plays a crucial role in suppressing seizures in experimental epilepsy models via modulating the CaMKII/NMDA receptor pathway, and miR-219 supplementation may be a potential anabolic strategy for ameliorating epilepsy. Furthermore, Qi et al. (2020) found that miR-494 overexpression could repress RIPK1, which inactivates the NF-κB signaling pathway, acceleration of cell proliferation, and suppression of apoptosis in hippocampal neurons of epileptic rats, attenuating neuronal injury and epilepsy development.

In mouse models of epilepsy, attempts have been made to control epilepsy by regulating the expression of miR-146a. Tao et al. (2017) found that intranasal delivery of miR-146a mimics could improve epilepsy onset and hippocampal damage in the acute phase of lithium-pilocarpine-induced epilepsy by modulating the expression of inflammatory factors. Intracerebroventricular injection of miR-146a could also relieve epilepsy in an immature rat model of lithium-pilocarpine-induced status epilepticus (Wang X. et al., 2018).

In general, multiple miRNAs are potential therapeutic targets for the treatment of epilepsy; however, there are still some challenges to their clinical application. First, previous studies have mostly been performed only in a single model or species. Thus, the results may need to be verified in models representing different etiologies or in larger animals. Secondly, it is necessary to understand the mechanism of miRNA-targeted therapy. However, the establishment of these mechanisms is limited to a small number of studies, and the mechanisms have rarely been verified *in vivo*. Thirdly, the safety of oligonucleotides that target brain miRNAs needs to be extensively evaluated in preclinical studies.

## Conclusion

Emerging studies have shown that miRNAs are key gene regulation factors in epilepsy pathogenesis. Indeed, miR-146a and miR-155 might be critical miRNAs involved in this disease. Expression differences of circulating miRNAs may be useful biomarkers for diagnosing, evaluating prognosis, and predicting treatment response. Differentially expressed miRNAs can be used to identify changes in the molecular structure and cellular pathways in epilepsy patients and represent possible treatment targets. However, the results of multiple studies on miRNA as biomarkers for epilepsy diagnosis need to be unified. Regulating pathological genes and interfering with other pathogenic mechanisms can produce therapeutic effects. Thus, the development of effective miRNA therapeutics holds great promise for potential therapeutic strategies for epilepsy.

## Author Contributions

JW and JZ drafted and revised the manuscript. JZ drafted and modified the figures and tables. Both authors approved the final version of the manuscript and agreed to be accountable for all aspects of the work to ensure that questions related to the accuracy or integrity of any part of the work are appropriately investigated and resolved.

## Conflict of Interest

The authors declare that the research was conducted in the absence of any commercial or financial relationships that could be construed as a potential conflict of interest.
