# Supplementary material for: MicroRNA Dysregulation in Epilepsy: From Pathogenetic Involvement to Diagnostic Biomarker and Therapeutic Agent Development
Source: Front Mol Neurosci. 2021 Mar 12;14:650372. doi: 10.3389/fnmol.2021.650372 (PMC7994516; doi:10.3389/fnmol.2021.650372)
Supplement: Supplementary file 1 [file Table_1.DOCX]

**Table 1.** Studies of diagnostic or prognostic biomarkers in epilepsy

| **MIRNAs** | **Expression Changes** | **Sample** | **Biomarker type and indication** | **References** |
| --- | --- | --- | --- | --- |
| miR-134 | Down-regulated | Plasma | Diagnosis biomarker for MTLE | Avansini et al., 2017 |
| miR-145, miR-181c, miR-199a, miR-1183 | Up-regulated | Blood | Diagnosis biomarker for MTLE with hippocampal sclerosis | Antônio et al., 2019 |
| miR-328-3p, miR-654-3p | Up-regulated | Serum | Diagnosis and surgical prognosis biomarker for MTLE with hippocampal sclerosis | Ioriatti et al., 2020 |
| miR-106b-5p | Up-regulated | Serum | Diagnosis biomarker for epilepsy | Wang et al., 2015b |
| miR-106b, miR-146a | Up-regulated | Serum | Diagnosis and severity biomarker for epilepsy | An et al., 2016 |
| miR-129-2-3p | Up-regulated | Plasma and temporal cortex | Diagnosis and severity biomarker for TLE | Sun et al., 2016b |
| miR-145-5p | Down-regulated | Plasma | Diagnosis and severity biomarker for TLE | Shen et al., 2019 |
| miR-30a | Up-regulated | Serum | Diagnosis and severity biomarker for epilepsy | Sun et al., 2016a |
| miR-4521 | Up-regulated | Brain tissue and serum | Diagnosis biomarker for FCD with refractory epilepsy | Wang et al., 2016b |
| miR-323a-5p | Up-regulated | Plasma and brain cortex | Diagnosis biomarker for FCD with refractory epilepsy | Che et al., 2017 |
| miR-146a, miR-155 | Up-regulated | Serum | Diagnosis biomarker for genetic generalized epilepsy | Martins-Ferreira et al., 2020 |
| miR-301a-3p | Up-regulated | Serum | Diagnosis biomarker for drug resistance epilepsy | Wang et al., 2015a |
| miR-134, miR-146a | Up-regulated | Serum | Diagnosis biomarker for drug resistance epilepsy | Leontariti et al., 2020 |

MTLE, mesial temporal lobe epilepsy; TLE, temporal lobe epilepsy; FCD, focal cortical dysplasia;
