# Supplementary material for: MicroRNA Dysregulation in Epilepsy: From Pathogenetic Involvement to Diagnostic Biomarker and Therapeutic Agent Development
Source: Front Mol Neurosci. 2021 Mar 12;14:650372. doi: 10.3389/fnmol.2021.650372 (PMC7994516; doi:10.3389/fnmol.2021.650372)
Supplement: Supplementary file 2 [file Table_2.DOCX]

**Table 2.** Summary of preclinical studies manipulating miRNAs in rodent models of epilepsy

| Studied miRNA | Alteration | Methods | Effect of manipulation on excitability/epilepsy | Affected target and/or pathway | References |
| --- | --- | --- | --- | --- | --- |
| miR-210 | Overexpression | Mimics | Reduction | VNS-induced neuroprotection against I/R injury | Jiang et al., 2015 |
| miR-137 | Overexpression | Mimics | Reduction | Inhibitory postsynaptic currents and presynaptic inhibitory neurotransmitter release | Wang et al., 2018a |
| miR-135a | Knockdown | Antagomirs | Reduction | Regulating Mef2 proteins | Vangoor et al., 2019 |
| miR-134 | Knockdown | Antagomirs | Reduction | Upregulating its target gene CREB | Gao et al., 2019 |
| miR-132 | Knockdown | Antagomirs | Reduction | miR-132/p250GAP/Cdc42 pathway | Yuan et al., 2016 |
| miR-204 | Overexpression | Mimics | Reduction | Regulating TrkB and ERK1/2-CREB signaling pathway | Xiang et al., 2016 |
| miR-219 | Overexpression | Mimics | Reduction | CaMKII/NMDA receptor pathway | Zheng et al., 2016 |
| miR-494 | Overexpression | Mimics | Reduction | Inactivation of the NF-κB signaling pathway | Qi et al., 2020 |
| miR-146a | Overexpression | Mimics | Reduction | Modulating expression inflammatory factors | Tao et al., 2017; Wang et al., 2018b |
